# Research on the evolutionary control of unsafe behavior of construction personnel based on multi-field coupled-homogeneous analysis model

**DOI:** 10.1371/journal.pone.0302263

**Published:** 2024-05-08

**Authors:** Haoran Zhao, Changfeng Wang, Qiushuang Zheng, Xuefeng Xia, Yanmin Ouyang

**Affiliations:** 1 School of Economics and Management, Beijing University of Posts and Telecommunications, Beijing, China; 2 China National Health Development Research Center, Beijing, China; 3 School of Economics, Peking University, Beijing, China; Nanjing Audit University, CHINA

## Abstract

Unsafe behavior among construction personnel poses significant risks in petroleum engineering construction projects. This study addresses this issue through the application of a multi-field coupled homogeneous analysis model. By conducting case analyses of petroleum engineering construction accidents and utilizing the WSR methodology, the influencing factors of unsafe behaviors among construction personnel are systematically categorized into organizational system factors, equipment management factors, and construction personnel factors. Subsequently, employing Risk coupling theory, the study delves into the analysis of these influencing factors, discussing their coupling mechanisms and classifications, and utilizing the N-K model to elucidate the coupling effect among them. Furthermore, a novel approach integrating coupling analysis and multi-agent modeling is employed to establish an evolutionary model of construction personnel’s unsafe behavior. The findings reveal that a two-factor control method, concurrently reinforcing equipment and construction personnel management, significantly mitigates unsafe behavior. This study provides valuable insights into the evolution of unsafe behavior among construction personnel and offers a robust theoretical framework for targeted interventions. Significantly, it bears practical implications for guiding safety management practices within petroleum engineering construction enterprises. By effectively controlling unsafe behaviors and implementing targeted safety interventions, it contributes to fostering sustainable development within the petroleum engineering construction industry.

## Introduction

Petroleum engineering construction is complex and high-risk, with unsafe behavior among construction workers being a leading cause of accidents. Various factors interact, resulting in frequent unsafe behavior. Analyzing these factors is crucial for risk management and prevention of accidents [1–3].

The study of risk management in petroleum engineering construction has primarily focused on risk identification, assessment, and control. Methods used for risk identification include qualitative and semi-quantitative methods such as expert assessment, hierarchical analysis, risk ranking matrices, and LCA methodology [4–7]. Various intelligent risk assessment methods have been proposed, including self-organizing mapping neural network theory, dynamic risk-based inspection methodology, and assessment models based on neural networks and Monte Carlo simulation [8–10]. Bayesian network models, hybrid fuzzy DEMATEL-ANP approaches and the PSO-SVR algorithm have been used for the quantitative assessment of petroleum engineering hazards [11–13]. For risk control, qualitative methods such as surveys, interviews, and case studies [14–16], as well as quantitative methods that combine risk control with mathematical models and artificial intelligence algorithms [17, 18], have been employed. However, research in this area has been limited in its examination of the coupling effects of multiple risk factors that can contribute to petroleum engineering construction accidents.

Risk coupling theory refers to a theoretical framework in the field of risk management that considers the interactions and influences between different risk factors. It emphasizes the potential interconnections and mutual impacts among various risk factors, which may lead to the occurrence of one risk event or exacerbate the occurrence of others, thereby increasing the overall risk level [19–21]. Several researchers have explored the coupling effects of multiple risk factors in various industries by constructing N-K models, including marine ship accidents and tunnelling construction accidents [22, 23]. These studies aim to achieve dynamic control of coupled risks. Combining the N-K model with different research methods has also been used to enhance the risk coupling model, such as the AHP-based N-K model for constructing a risk coupling model of transportation accidents in complex marine environments [24]. The BBN-NK model has been utilized for risk analysis of tunnel fire accidents, quantifying various risk factors and simulating the frequency of tunnel fire accidents [25]. Furthermore, the N-K model has been applied to construct a social risk coupling evaluation model for major projects based on complex network theory [26], as well as a risk coupling method for offshore oil well accidents using the Dynamic Bayesian Network (DBN) and N-K model to analyze the interaction among risk factors and the accident risk evolution process [27]. While the N-K model is extensively used in engineering risk management, there is a lack of research on analyzing the coupling effects of factors impacting the unsafe behavior of personnel involved in petroleum engineering construction using the N-K model. Fundamentally, the overarching objective of risk coupling theory is to facilitate a comprehensive comprehension and adept management of the intricate relationships inherent within risk systems [28]. By doing so, it endeavors to empower stakeholders with the tools necessary for more efficacious prevention and mitigation strategies across a spectrum of risks. Therefore, this research employs risk coupling theory to elucidate the evolutionary process of unsafe behaviors among construction personnel within the complex system of petroleum engineering construction projects. This aids in gaining a deeper understanding of the interactions among various factors influencing unsafe behaviors of construction personnel and provides a theoretical framework for constructing subsequent risk evolution model.

Unsafe behavior of construction personnel is a fundamental component of the petroleum engineering construction system that significantly impacts engineering construction risks. Multi-Agent modelling has emerged as an effective approach to linking the behavior of micro-level subjects in complex systems with macro-level problems, and to assess and reveal the evolution of management issues based on the behavior of micro-level subjects [29, 30]. Building an evolutionary model of unsafe behavior of engineering construction personnel using multi-agent technology can reproduce the evolutionary process of unsafe behavior and quantify the construction risk status, and can also combine with risk control strategies to control construction risks by intervening in unsafe behavior. This approach forms a comprehensive construction risk control method for petroleum engineering. Prior research has employed multi-agent technology to investigate engineering risks, including the identification of the main risk factors of shield tunnelling projects using a safety computational experiment system and the simulation of various risk control strategies for construction projects using a risk evolution model based on multi-agent modelling and stochastic methods [31, 32]. Multi-agent-based collaborative emergency decision-making algorithms have also been proposed for emergency response to traffic accidents, and simulation modeling of human-machine-environment-related risk factors in coal mines has been conducted using multi-agent technology [33, 34].

In summary, in the realm of existing research concerning the analysis of unsafe behaviors among construction personnel in petroleum engineering construction, there remains a scarcity of studies that amalgamate macro-level influencing factors with the micro-level evolution of these behaviors. Gaps persist in the research pertaining to the control of unsafe behaviors among construction personnel based on the risk coupling theory. Hence, this study explores the coupling mechanism and effect between the influencing factors of unsafe behaviors by combining the WSR methodology and the N-K model. Additionally, it proposes a novel multi-field coupled homogeneous analysis model by incorporating the multi-agent modeling method to elucidate the evolution mechanism of unsafe behaviors among construction personnel. By dynamically scrutinizing the evolution of construction personnel’s unsafe behaviors, this study formulates targeted safety interventions aimed at addressing the issue of unsafe behaviors in petroleum engineering construction projects. These innovative methodologies contribute to a more profound comprehension of the evolution of unsafe behavior among construction personnel and offer valuable insights for enhancing safety in petroleum engineering construction endeavors.

## Coupling analysis of factors affecting unsafe behavior of construction personnel

Through an analysis of construction accident statistics in petroleum engineering companies, coupled with the application of the WSR methodology, this study delves into the factors influencing unsafe behaviors across three distinct levels: physical, principle, and human factors. It elucidates the interaction and coupling mechanisms among these factors. Furthermore, leveraging the risk coupling theory, the study categorizes influencing factors into single-factor coupling, two-factor coupling, and multi-factor coupling. Subsequently, employing the N-K model, a probability analysis of various coupling forms is conducted. These efforts aim to furnish a theoretical framework for the construction of a multi-agent model of unsafe behavior evolution.

### Identification of influencing factors

This paper counts 96 cases of construction accidents in petroleum engineering companies. The sources of case statistics are: government websites, published accident investigation reports and "Cases of Petroleum Engineering Safety Accidents". By analyzing the causes of unsafe behavior of construction workers in different construction accident cases, the influencing factors of unsafe behavior of construction workers can be derived.

In conjunction with a case study of construction accidents and the WSR methodology, the factors that influence the unsafe behavior of construction workers are summarized. Professor Gu, a renowned Chinese systems science expert, suggested the "Wuli-Shili-Renli" (WSR) methodology as a comprehensive system methodology [35], which is based on the principle of analyzing problems from multiple perspectives in order to resolve problems in complex systems more effectively. The WSR methodology splits complex systems into three levels: physical level, principle level, and human level. The physical level refers to the system’s matter and energy, the principle level refers to the system’s logic and information, and the human level refers to the system’s human behavior and decision-making. Applying WSR methodology to petroleum engineering construction risk cases, analyzing the causes of unsafe behaviors of construction workers individually, and discovering that three factors influence construction personnel’s unsafe behavior: equipment management, organizational system, and construction personnel, which correspond to the physical level, the principle level, and the human level, respectively.

The causes of unsafe behavior of construction personnel in each case were studied based on the three levels of unsafe behavior influencing factors, and the statistical distribution of factors influencing unsafe behavior of petroleum engineering construction personnel is determined (Table 1). The physical factor-principle factor effect implies that the unsafe behavior of construction workers is the result of the interaction between equipment management factor and organization system factor, and the rest are the same.

**Table 1:** Statistical table of influencing factors causing unsafe behavior of construction personnel.

Type of Effect	Causes	Number of Unsafe Behavior
Single-factor Effect	Physical Factor	2
	Principle Factor	5
	Human Factor	8
Two-factor Effect	Physical Factor-Principle Factor	16
	Physical Factor- Human Factor	24
	Principle Factor -Human Factor	35
Multi-factor Effect	Physical Factor-Principle Factor- Human Factor	6

### Coupling mechanism of unsafe behavior influencing factors

**Fig 1:**
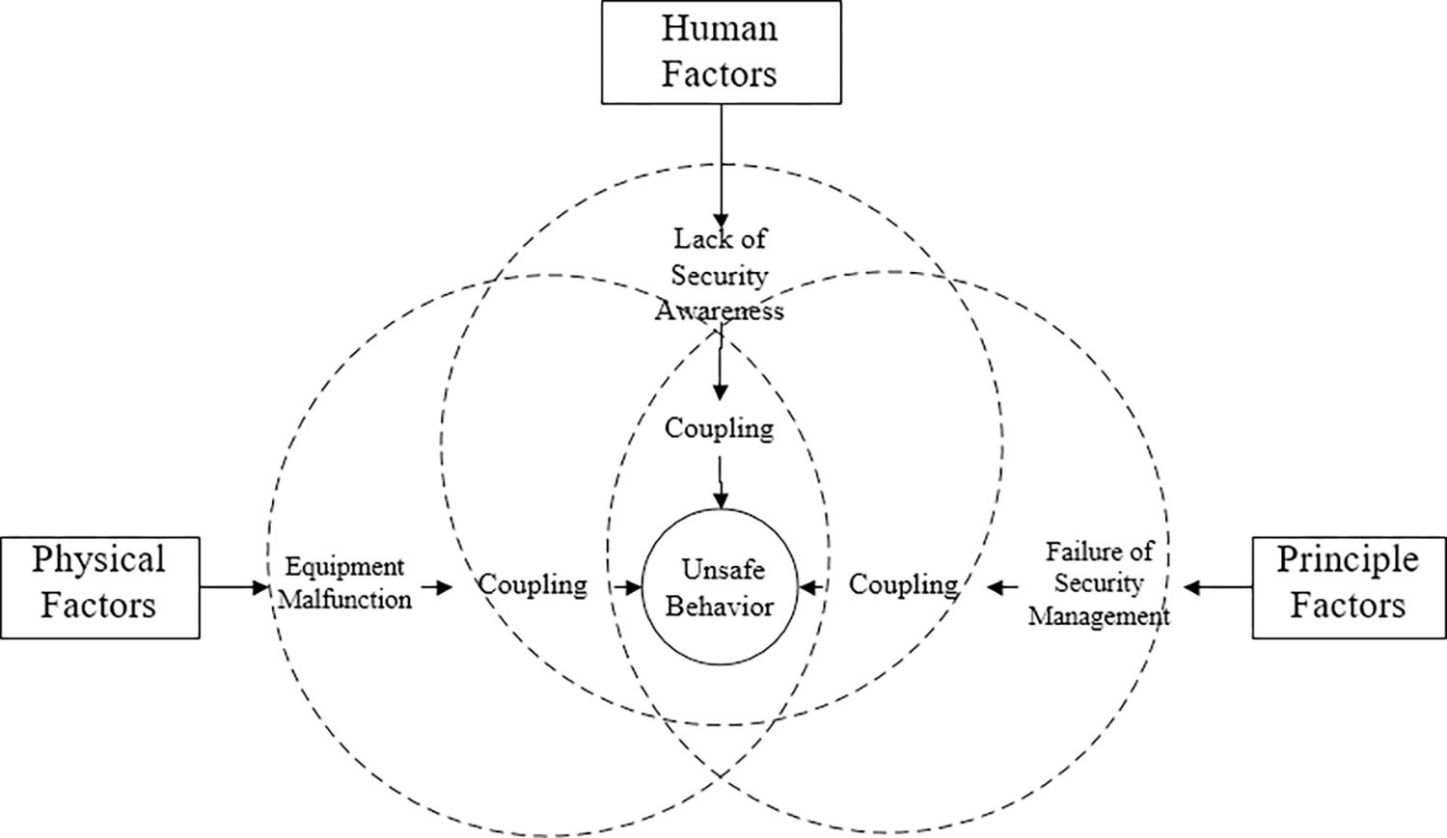
Multidimensional coupling mechanism of factors influencing unsafe behavior of construction personnel.

Coupling refers to the degree or manner in which two or more systems, components, or subsystems interact or influence each other, whereas risk coupling refers to the interaction or interdependence between two or more risk events in a risk system, where the occurrence of one risk event may cause the occurrence or exacerbation of other risk events, thereby increasing the overall risk level of the system or project. Hence, the coupling of factors influencing unsafe behaviors of construction workers in petroleum engineering refers to the mutual influence and interdependence among the factors that affect construction workers’ unsafe behaviors. As seen in Fig 1, the coupling mechanism of elements influencing risky behavior in construction workers is investigated from the three-dimensional perspective of physical factor, principle factor, and human factor.

Fig 1 reveals that the influence of physical level factor may result in the failure of construction equipment; the influence of principle level factor may result in the failure of the organization’s management system and the decline of the safety atmosphere; and the influence of human level factor may result in the lack of safety awareness among construction personnel. The coupling of these three factors leads to the unsafe conduct of construction workers. In the process of petroleum engineering construction, if the effect of one of the aforementioned factors does not reach the threshold of unsafe behavior of construction personnel, it will not directly lead to the occurrence of unsafe construction behavior. However, the interaction of the aforementioned factors will produce a coupling effect, which will increase the likelihood that construction personnel will engage in unsafe behavior.

### Coupling types of unsafe behavior influences

The risk coupling can be categorized into the three following categories based on the number of factors that influence the risky behavior of construction professionals involved in the coupling.

1. Single-factor coupling, refers to the internal interaction of certain factors that affect construction workers to implement unsafe behaviors. There are three types of single factor coupling, namely equipment management factor coupling, organizational system factor coupling, and construction personnel factor coupling.
2. Two-factor coupling, refers to the interaction between two types of factors that affect construction personnel to implement unsafe behaviors. There are three types of two factors coupling, namely equipment management-organizational system factors coupling, equipment management-construction personnel factors coupling, organization system- construction personnel factors coupling.
3. Multi-factor coupling, refers to the interaction among three or more factors that affect construction personnel to implement unsafe behaviors. There is one type of multi-factor risk coupling, which is the coupling of equipment management-organization system-construction personnel factors.

### Coupling analysis of factors influencing unsafe behavior based on N-K model

In recent years, risk coupling models have been increasingly investigated by academics. The N-K model, which is a general model used to represent interactions in complex systems, is suited for examining the coupling between factors that influence the unsafe behavior of construction workers.

Professor Kauffman proposed the N-K model for the analysis of gene combinations in the 1990s based on random Boolean networks [36]. The N-K model describes the system as a network of N elements, each having two states (0 or 1), and K connections between the elements that represent their interactions and dependencies, the minimum value of K is 0, and the maximum value is N-1.

The evolutionary system of risky construction worker behavior consists of three distinct types of influencing factors: equipment management factor, organizational system factor, and construction personnel factor. According to the statistics of 96 petroleum engineering construction accident incidents in Table 1, "0" and "1" are used to indicate the three categories of influencing elements. "0" indicates that the risk factor is not involved in coupling but construction workers still engage in unsafe behavior and "1" indicates that the risk factor is involved in coupling and causes construction workers to engage in unsafe behavior, then there are eight distinct forms of coupling between the three influencing factors.

**Fig 2:**
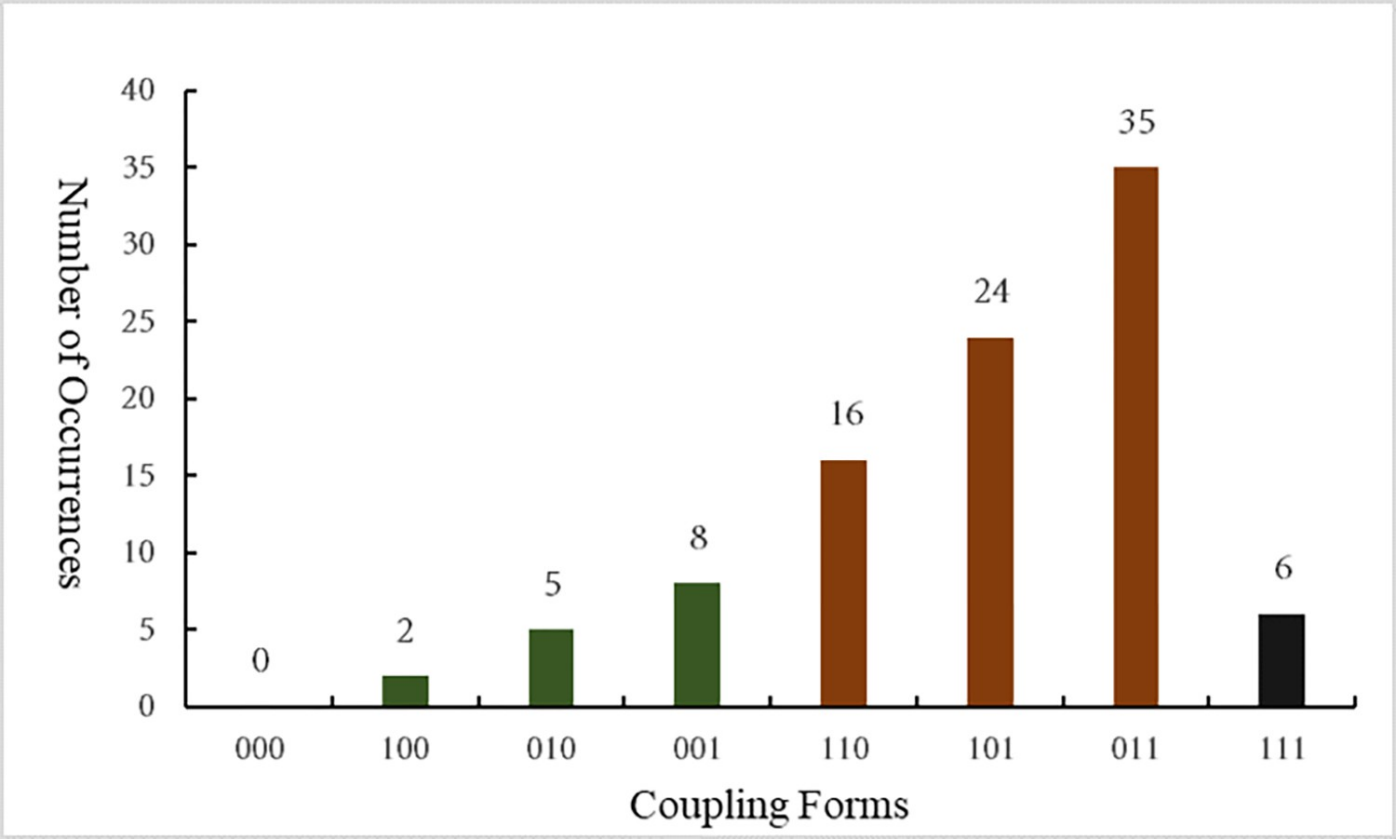
Statistics of the number of unsafe behaviors of construction personnel under different coupling forms.

For example, the coupling form "110" represents the mutual coupling of equipment management and organizational system factors that lead to unsafe behaviors among construction workers, whereas "P_110_" represents the probability that the mutual coupling of equipment management and organizational system factors leads to unsafe behaviors among construction workers. The number and probability of different coupling forms leading to unsafe behavior of construction personnel are shown in Fig 2 and Table 2, respectively.

**Table 2:** Probability of unsafe behaviors of construction personnel under different coupling forms.

**Single-factor Coupling**	Coupling Forms	000	100	010	001
	Occurrence Probability	P_000_ = 0	P_100_ = 0.021	P_010_ = 0.052	P_001_ = 0.083
**Two-factor Coupling**	Coupling Forms	110	101	011	—
	Occurrence Probability	P_110_ = 0.167	P_101_ = 0.25	P_011_ = 0.365	—
**Multi-factor Coupling**	Coupling Forms	111	—		
	Occurrence Probability	P_111_ = 0.062	—		

In Table 2, P_110_ = 0.167 indicates that the probability of construction personnel exhibiting unsafe behaviors due to the mutual coupling of equipment management and organizational system factors is 0.167, which is the ratio of the number of times equipment management and organizational system factors cause construction workers to exhibit unsafe behaviors to the total number of unsafe behaviors exhibited by construction workers. Through similar calculations, it is possible to determine the probability of construction workers engaging in unsafe behavior under various coupling forms. The probabilistic analysis of the diverse forms of coupling derived from the N-K model can aid in parameterizing the state transition process for construction workers in subsequent multi-agent model. Risk coupling precipitates the manifestation of unsafe behaviors, constituting a transformation in the state of construction workers. Thus, the probability of unsafe behavior resulting from various forms of risk coupling serves as the probability of the state transition process occurrence in the multi-agent model.

## Evolution model of unsafe behavior of construction personnel

Beginning with the control of unsafe construction worker behaviors, the relationship between the influencing factors of unsafe behaviors and the status of construction workers is investigated. In conjunction with the multi-agent modeling method and the coupling analysis of unsafe behavior affecting elements, a Multi-Agent model of unsafe behavior evolution is devised based on the coupling of influencing factors.

### Related settings of unsafe behavior evolution model

The establishment of the evolution model of construction workers’ unsafe behavior based on multi-agent necessitates the selection of a suitable modeling platform and the establishment of an appropriate engineering context and system environment.

**(1) Modeling platform.** AnyLogic not only provides a visual interface and graphical modeling tools, but also enables visual analysis of systems to aid users in comprehending the behavior and attributes of multi-intelligent systems, which are widely utilized in management science study disciplines. In this research, all multi-agent modeling and simulation experiments are implemented on the Anylogic8 platform.
**(2) Engineering background.** Using petroleum engineering as an illustration, the primary source of its construction risk is the unsafe behavior of construction personnel.
**(3) System environment setting.** The model represents the state of petroleum engineering construction risk via changes in the behavior of construction personnel, and the fundamental unit of the model is the construction personnel. The equipment management, organizational system, and construction personnel factors are taken as the influencing factors of the construction personnel’s behavior, and the control parameters are set to represent the strength of the control measures of the influencing factors. The system environment setting contains the following:
  ① Number of construction personnel and simulation cycle;
  ② Construction personnel in various states: include the safe, transitional, and unsafe states of construction personnel;
  ③ Ways and degrees of influence of unsafe behavior influencing factors on the state of construction personnel;
  ④ Ways and degrees of influence of control measures on the state of construction personnel;
  ⑤ Behavioral decision making and evolutionary mechanisms: describe the behavioral transformations and transitional pathways of construction personnel in response to varying conditions.
**(4) Setting of construction personnel behavior status transformation.** By analyzing the evolution process of unsafe behavior of construction workers in construction accident cases, it is set that in the initial stage of system evolution, construction workers are performing safe construction, called normal status.

When the construction personnel are affected by organizational system factors, their state will undergo two transitions: transition1: from a normal state to a state that produces unsafe behavior, called an unsafe status; transition2: from a normal state to a state that is affected by the organization system but does not produce unsafe behavior, called transition status1.

Afterwards, when the construction personnel are affected by equipment management factors, their state will undergo three transitions: transition3: only affected by equipment management factors, from transition status1 to an unsafe status; transition4: affected by the coupling effect of organizational system factors and equipment management factors, from transition status1 to unsafe status, transition5: transition from transition status1 to a state affected by equipment management factors but without unsafe behavior, called transition status2.

Finally, when construction personnel are affected by construction personnel factors, their state will undergo four transitions: transition6: only affected by construction personnel factors, from transition status2 to an unsafe status, transition7: affected by the coupling of equipment management factors and construction personnel factors Influenced from transition status2 to unsafe status, transition8: affected by the coupling effect of organizational system factors and construction personnel factors, from transition status2 to unsafe status, transition9: affected by three factors of organizational system, equipment management and construction personnel Coupling effects, from transition status2 to unsafe status.

**Fig 3:**
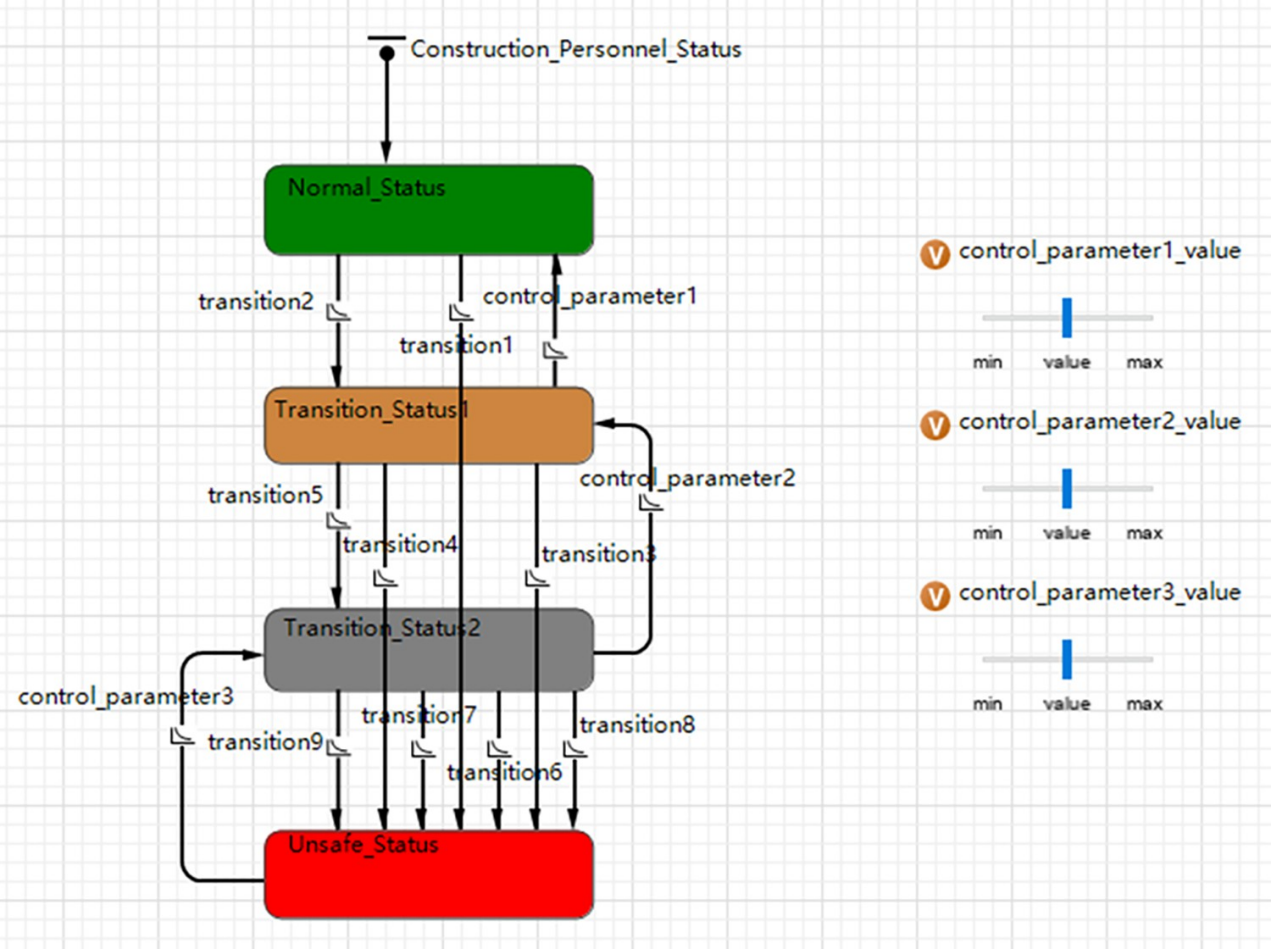
Evolution model of unsafe behavior of construction personnel.

As depicted in Fig 3, create an evolution model of unsafe construction personnel behavior using the AnyLogic platform.

**(5) Model variables setting.** Expert evaluation method and entropy method are used to determine the comprehensive weight of influencing factors in order to quantify the importance of three types of influencing factors, namely equipment management, organization system, and construction personnel. Following are the specific steps of the entropy method for calculating comprehensive weights:
  ① Collecting data via expert evaluations, constructing evaluation matrices, and normalizing;
  ② Construct standardized matrix:

$$P_{ij}=\frac{X_{ij}}{\sum_{i=1}^{n}X_{ij}}$$ (1)

③ Calculate the entropy value of each indicator:

$$\alpha_{j}=-\frac{1}{\ln n}\sum_{i=1}^{n}P_{ij}\ln P_{ij}$$ (2)

④ Calculate the coefficient of difference:

$$Z_{j}=1-\alpha_{j}$$ (3)

⑤ Calculate the weight:

$$W_{j}=\frac{Z_{j}}{\sum_{i=1}^{n}Z_{j}}0\leq W_{j}\leq 1,\sum_{j=1}^{m}W_{j}=1$$ (4)

Using entropy method to calculate the comprehensive weight of the three types of influencing factors, as shown in Table 3.

**Table 3:** Comprehensive weight of factors influencing unsafe behavior.

	Equipment Management	Organizational System	Construction Personnel
**Combined Weights**	0.171	0.303	0.526

The model’s variables and parameters are set as shown in Table 4 based on the previous coupling analysis of the state evolution process of construction workers.

**Table 4:** Multi-agent model variables settings.

No.	Variables	Connotation
1	Normal Status	The status of construction personnel implementing safe behaviors
2	Transition Status1	The status of construction personnel affected by organizational system factors but not producing unsafe behaviors
3	Transition Status2	The status of construction personnel affected by equipment management factors but not producing unsafe behaviors
4	Unsafe Status	The status of construction personnel implementing unsafe behaviors
5	Transition1	The probability that construction personnel is transformed into unsafe status by organizational system factors, based on the previous coupling analysis, the value is set to P_010_ = 0.052
6	Transition2	The probability that construction personnel is transformed into transition status1 by organizational system factors, the value is 1- P_010_ = 0.948
7	Transition3	The probability that construction personnel is transformed into unsafe status by equipment management factors, based on the previous coupling analysis, the value is set to P_100_ = 0.021
8	Transition4	The probability that construction personnel is transformed into unsafe status by the coupling effect of organizational system and equipment management factors, based on the previous coupling analysis, the value is set to P_110_ = 0.167
9	Transition5	The probability that construction personnel is transformed into transition status2 by equipment management factors, the value is 1- P_100_- P_110_ = 0.812
10	Transition6	The probability that construction personnel is transformed into unsafe status by construction personnel factors, based on the previous coupling analysis, the value is set to P_001_ = 0.083
11	Transition7	The probability that construction personnel is transformed into unsafe status by the coupling effect of equipment management and construction personnel factors, based on the previous coupling analysis, the value is set to P_101_ = 0.25
12	Transition8	The probability that construction personnel is transformed into unsafe status by the coupling effect of organizational system and construction personnel factors, based on the previous coupling analysis, the value is set to P_011_ = 0.365
13	Transition9	The probability that construction personnel is transformed into unsafe status by the coupling effect of three factors, based on the previous coupling analysis, the value is set to P_111_ = 0.062
14	Control Parameter1	Parameter controlling the organizational system factor, with the initial value set to 1
15	Control Parameter2	Parameter controlling the equipment management factor, with the initial value set to 1
16	Control Parameter3	Parameter controlling the construction personnel factor, with the initial value set to 1

### Initial setting of model parameters and evolutionary results

**Fig 4:**
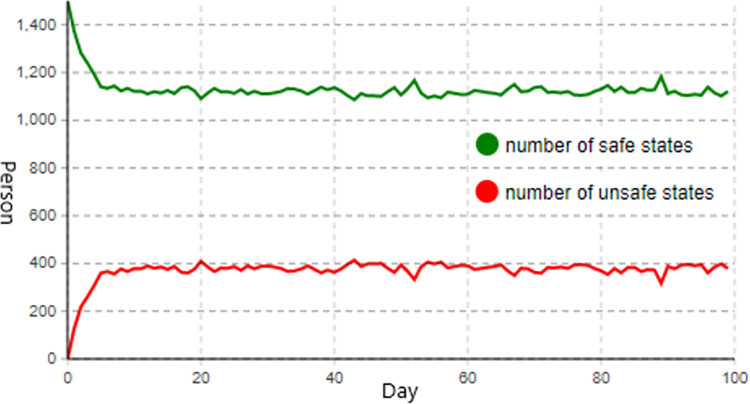
Construction personnel status change diagram under initial setting.

Referring to the current state of petroleum engineering construction enterprises and the general effectiveness of their control measures for the three influential factors of organizational structure, equipment management, and construction personnel. In the initial setting, the total number of construction workers is set to 1,500, the values of control parameter1, control parameter2, and control parameter3 are all set to 1, and the number of simulation days is set to 100. The number of construction workers in the normal, transition 1, and transition 2 states is collectively referred to as the number of safe states, while the number of construction workers in an unsafe status is referred to as the number of unsafe states. Fig 4 depicts the construction personnel state change diagram generated by executing the unsafe behavior evolution model of construction personnel.

As depicted in Fig 4, under the initial setting, the number of construction personnel transforming into an unsafe state increased due to the influence of unsafe behavior influencing factors. When the number reaches about 380, the rising trend of the number of construction personnel in an unsafe state slows and reaches a stable state as a result of construction enterprises’ control measures. When the system reaches a stable state, the number of construction personnel in an unsafe state fluctuates considerably and will remain close to 400, representing nearly one-third of the total number of workers, indicating that the construction risk of petroleum engineering is relatively high at this time. Consequently, after adjusting the control parameters, the results of the evolution of unsafe behavior of construction personnel under the conditions of single-factor, two-factor, and multi-factor control are studied, analyzed, and summarized to identify key measures to control unsafe behaviors of construction personnel.

## Result analysis by controlling different influencing factors

Under the assumption that the sum of the control strengths of the influencing factors is the same, the evolution process of the unsafe behavior of construction personnel is simulated when controlling a single factor, a double factor, and a multi-factor, and the number of people in an unsafe status when the system is stable is analyzed to compare the effects of various control methods.

### Single-factor control

**Fig 5:**
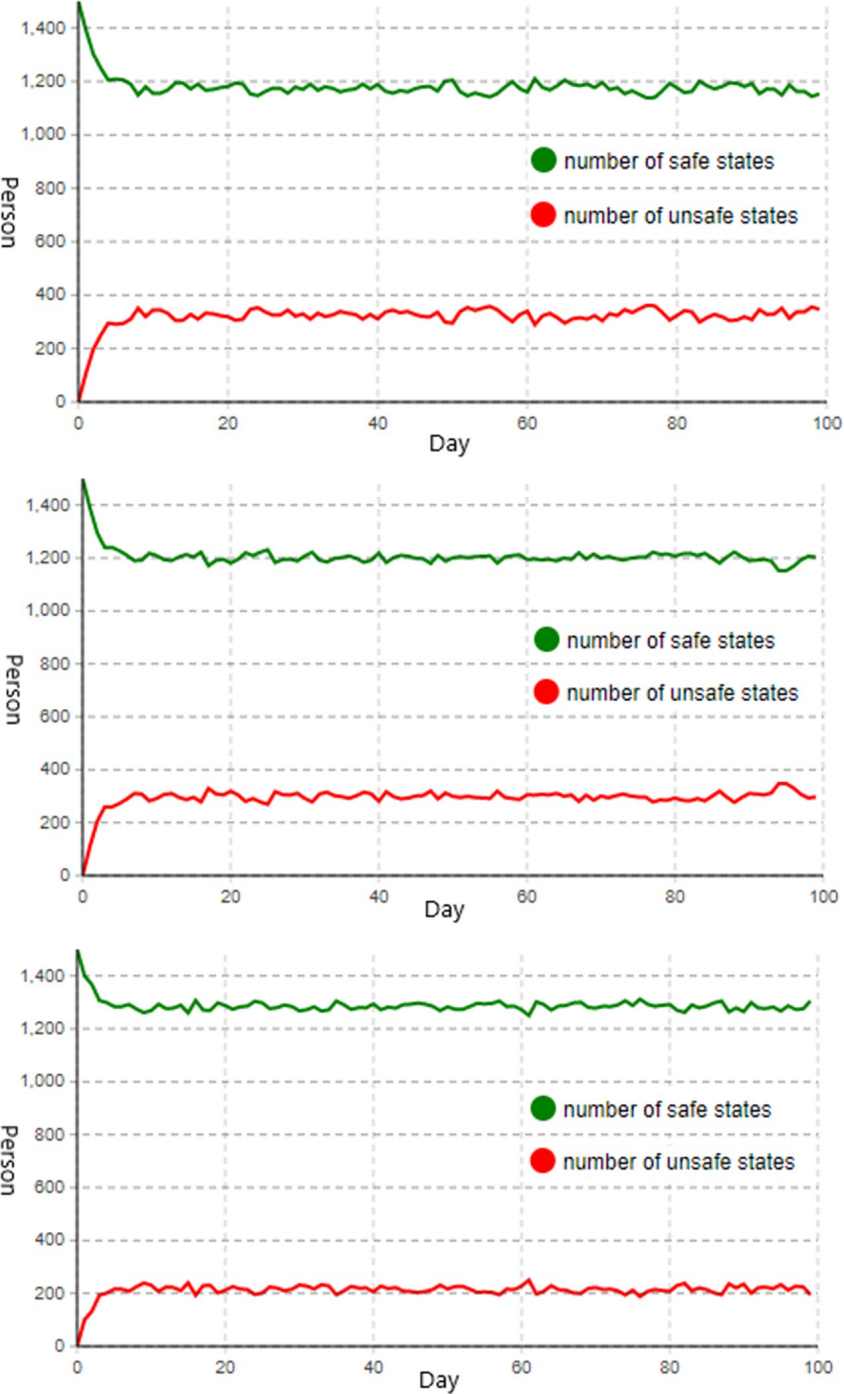
Diagram of construction personnel status change. **(a)** when controlling organizational system factor; **(b)** when controlling equipment management factor; **(c)** when controlling construction personnel factor.

**(1) Strengthen the control of organizational system factor.** Assuming that the control strength of a single factor is increased by 1 unit to simulate the strengthening of the management of organizational system factor, the value of control parameter1 is increased to 2, while control parameters2 and control parameters3 maintain their initial values. Analyzing the influence on the behavioral state of construction personnel when strengthening the control of organizational system factor yields the construction personnel status change diagram in Fig 5(A) below.

As depicted in Fig 5(A), when only reinforcing the control of organizational system factors, the number of construction personnel in an unsafe state slows after increasing to about 300 as a result of the strengthening of control measures and reaches a stable state. When the system reaches a stable state, the number of construction workers in an unsafe condition will remain at approximately 350. At this point, not only will the number of unsafe workers fluctuate more, but the decline relative to the initial setting will be minimal, indicating that the control effect does not increase significantly.

**(2) Strengthen the control of equipment management factor.** Assuming that the control strength of a single factor is increased by 1 unit to simulate the strengthening of the management of equipment management factor, the value of control parameter2 is increased to 2, while the values of control parameter1 and control parameter3 maintain their initial values. Analyzing the influence on the behavioral state of construction personnel when strengthening the control of equipment management factor yields the construction personnel status change diagram in Fig 5(B) below.

Fig 5(B) demonstrates that when only the equipment management control factor is reinforced, the number of construction workers in an unsafe state slows after reaching about 250 and then stabilizes as a result of the control measure’s strengthening. When the system reaches a stable state, the number of unsafe employees will remain around 300, representing one-fifth of the total number, and the fluctuation will decrease, indicating that the control effect has increased compared with the previous control method.

**(3) Strengthen the control of construction personnel factor.** Assuming that the control strength of a single factor is increased by 1 unit to simulate the situation of strengthening the management of construction personnel factor, the value of control parameter3 is increased to 2, while the values of control parameter1 and control parameter2 maintain their initial values. Analyzing the influence on the behavioral state of construction personnel when enhancing the control of the construction personnel factor yields the construction personnel status change diagram in Fig 5(C) below.

Fig 5(C) demonstrates that when only the construction personnel factor control is reinforced, the number of construction personnel in an unsafe state decelerates after reaching about 200 and reaches a stable state as a result of the strengthened control measure. When the system reaches its steady state, the number of unsafe employees will remain between 200 and 250, or one-sixth of the total. The number of unsafe workers fluctuates minimally at this time and decreases more than with the previous two control methods. Consequently, the control effect of this method is most apparent when only a single factor is controlled.

### Two-factor control

**(1) Strengthen the control of organizational system and equipment management factors.** Assuming that the control of the two types of factors increases by 0.5 units each, simulate the concurrent strengthening of the control of equipment management and organizational system factors.

**Fig 6:**
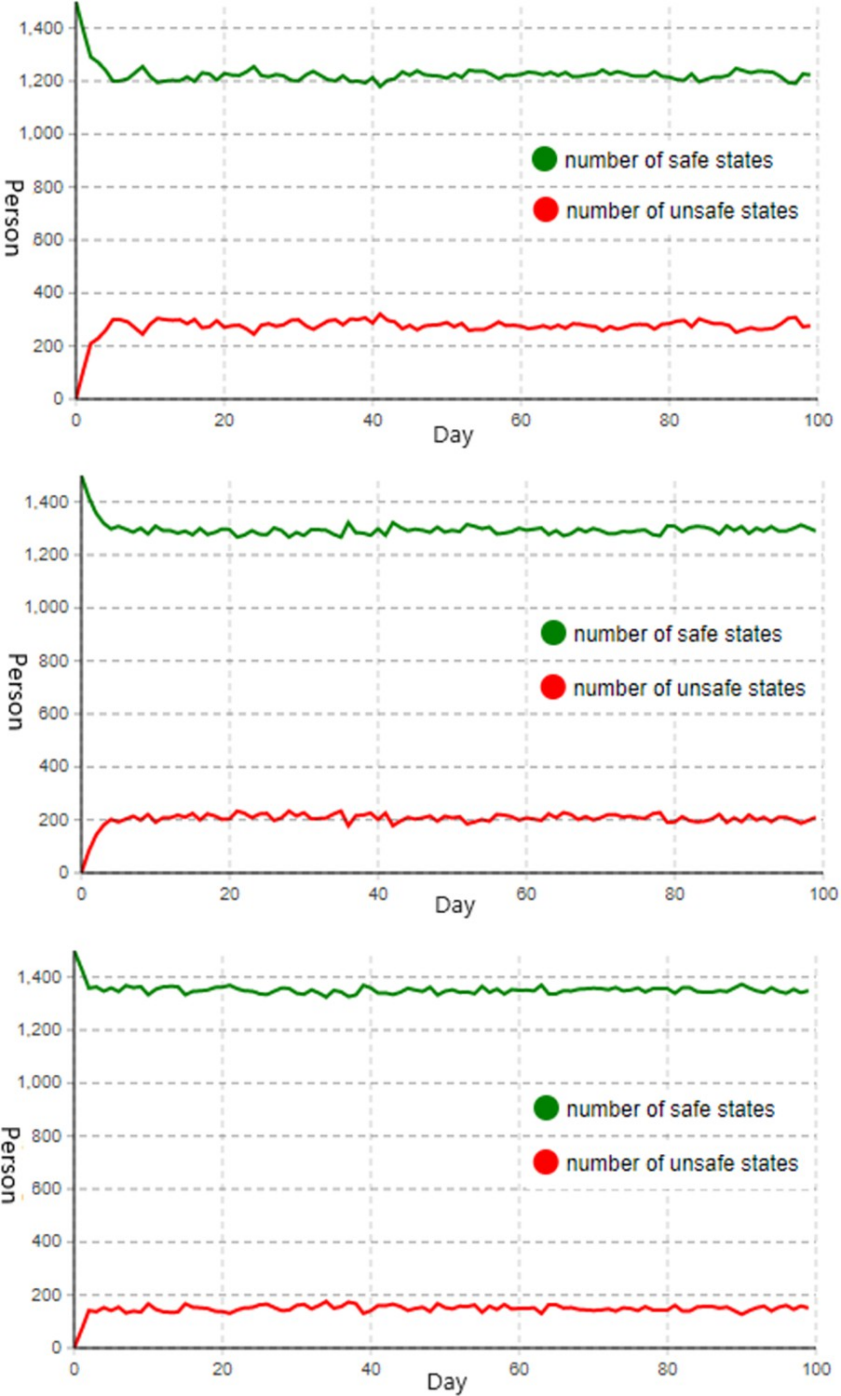
Diagram of construction personnel status change. **(a)** when controlling organizational system and equipment management factors; **(b)** when controlling organizational system and construction personnel factors; **(c)** when controlling equipment management and construction personnel factors.

Taking into account the coupling effect between the influencing factors and the ratio of the equipment management factor to the organizational system factor’s weight is 0.171:0.303. Consequently, the value of control parameter2 is increased to 1.5, the value of control parameter1 is increased to 1.885, and the initial value of control parameter3 is maintained. As shown in Fig 6(A) below, the status change diagram of construction personnel is obtained.

Fig 6(A) demonstrates that when the organizational system and equipment management factors control are simultaneously reinforced, the number of construction personnel in an unsafe state decelerates after reaching about 250 and then stabilizes as a result of the strengthened control measures. When the system is in a stable state, the number of construction employees in an unsafe state will fluctuate widely between 250 and 350, representing one-fifth of the total number. Therefore, compared to single-factor control, not only is the control effect not substantially improved, but it is also inferior to the effect of merely strengthening the control of construction personnel factors.

**(2) Strengthen the control of organizational system and construction personnel factors.** Assuming that the control of the two types of factors increases by 0.5 units each, simulate the concurrent strengthening of the control of organizational system and construction personnel factors.

Taking into account the coupling effect between the influential factors and the weight ratio of the organizational system factor to the construction personnel factor is 0.303:0.526. Consequently, the value of control parameter1 is increased to 1.5, the value of control parameter3 is increased to 1.868, and the initial value of control parameter2 is maintained. As shown in Fig 6(B) below, the status change diagram of construction personnel is obtained.

Fig 6(B) demonstrates that, when the organizational system and construction personnel factor control are simultaneously reinforced, the number of construction personnel in an unsafe state decelerates after reaching about 180 and then stabilizes due to the strengthening of control measures. When the system is in a stable state, the number of unsafe workers will be maintained at approximately 200, representing about one-seventh of the total number, with minor fluctuations. Consequently, its control effect is enhanced compared to the previous two-factor control method and is also superior to single-factor control.

**(3) Strengthen the control of equipment management and construction personnel factors.** Assuming that the control of the two types of factors increases by 0.5 units each, simulate the concurrent strengthening of the control of equipment management and construction personnel factors.

Taking into account the coupling effect between the influential factors and the weight ratio of the equipment management factor to the construction personnel factor is 0.171:0.526. Consequently, the value of control parameter2 is increased to 1.5, the value of control parameter3 is increased to 2.538, and the initial value of control parameter1 is maintained. The construction personnel status change diagram is obtained, as shown in Fig 6(C) below.

Fig 6(C) demonstrates that, when equipment management and construction personnel factors control are simultaneously strengthened, the number of construction personnel in an unsafe condition decreases gradually after reaching about 180 and then stabilizes as a result of the strengthened control measures. When the system is in a stable state, the fluctuation magnitude will be modest and the number of construction workers in unsafe conditions will be maintained at approximately 150. Among the three methods of implementing two-factor control, this method has the lowest number of construction workers in an unsafe state and the most obvious control effect.

### Multi-factor control

Assuming that the control of each type of factor increases by 0.33 units, simulate the situation where the management of organizational system, equipment management, and construction personnel factors are simultaneously strengthened.

**Fig 7:**
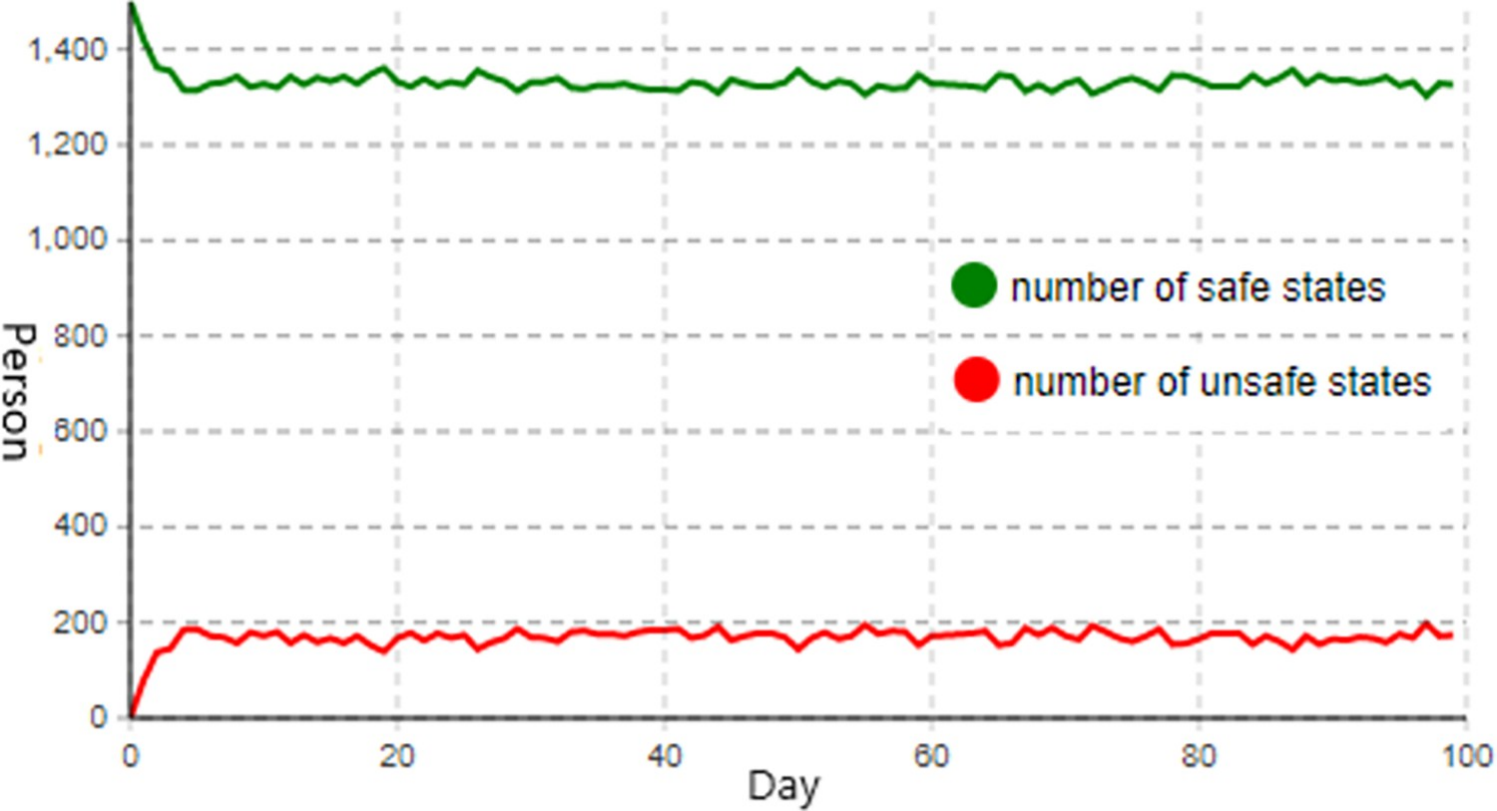
Diagram of construction personnel status change when controlling multi-factor.

Taking into account the coupling effect between the influential factors and the weight ratio of the three influential factor types is 0.171:0.303:0.526. Consequently, the values of control parameter1 are increased to 1.585, control parameter2 is increased to 1.33, and control parameter3 is increased to 2.015. As shown in Fig 7 below, the status change diagram of construction personnel is obtained.

As shown in Fig 7, the number of construction personnel in an unsafe state decelerates after reaching approximately 150 and reaches a steady state as a result of the strengthening of control measures by simultaneously enhancing the control of three influencing factors. When the system is in a stable state, there will be between 180 and 200 construction workers in unsafe conditions. Therefore, when compared to single-factor control, the control effect is significantly enhanced; however, when compared to two-factor control, the improvement in control effect is less than when equipment management and construction personnel factor controls are strengthened simultaneously, and the fluctuation magnitude is also larger. This indicates that under certain conditions, three-factor control is less effective than two-factor control.

### Discussion

In this chapter, the impact of different control strategies on the development of unsafe behaviors among construction workers was investigated through simulations employing single-factor, two-factor, and multi-factor control methods. While the single-factor approach demonstrates some efficacy in mitigating unsafe behaviors, its failure to account for the intricate interactions and coupling effects among different factors restricts its overall effectiveness, rendering it less potent compared to multi-factor interventions. Multi-factor control strategies offer a more holistic perspective by considering the interplay between diverse factors. However, given the typical constraints on resources for safety interventions in practical settings, the experiments compare the effectiveness of different multi-factor control methods under the constraint of constant total control intensity across influencing factors. The experimental results reveal that the two-factor control method, which concurrently enhances management of both equipment and construction personnel factors, emerges as the most efficient, significantly reducing the incidence of unsafe behaviors.

These findings offer crucial insights into risk management and safety interventions in the context of petroleum engineering construction projects. Particularly, regarding the formulation of precise and practical interventions, a comparative analysis of various multifactorial control methods is conducted to determine the optimal control method for addressing the unsafe behaviors exhibited by construction personnel, especially under resource-constrained conditions. Meanwhile, by establishing a multi-field coupled-homogeneous analysis model, this study delves into the underlying dynamics of unsafe behavior progression during petroleum engineering projects. It furnishes a theoretical framework to inform the safety management practices concerning project personnel, thereby contributing to the broader discourse on safety enhancement within construction domains.

## Research conclusion and future perspective

### Conclusion

The risk coupling theory is added to the evolution analysis of unsafe construction worker behaviors in petroleum engineering in this study. First, the classification and coupling mechanism of factors influencing unsafe behaviors of construction workers are discussed. Next, the coupling model of the factors influencing unsafe behavior is constructed using N-K model to reveal the coupling effect between various influencing factors. Lastly, the evolution model of unsafe behavior of construction personnel based on influencing factors coupling is constructed using multi-agent modeling, and the following conclusions are drawn:

1. Based on the case study and WSR methodology, the influencing factors of unsafe behavior of petroleum engineering construction personnel can be categorized as equipment management, organizational system, and construction personnel. There will be interaction and interdependence between the three influencing factors, thereby producing a coupling effect.
2. The coupling effect of multiple factors is the root cause of construction workers’ unsafe behaviors. There are potential factors influencing unsafe behavior in the process of petroleum engineering construction, and the likelihood of unsafe behavior of construction personnel due to the role of a single factor is low. However, when coupled with other influencing factors, there will be a coupling effect, and the likelihood of unsafe behavior of construction personnel will be greatly increased, resulting in construction accidents.
3. Using the coupling analysis of the influencing factors of construction personnel’s unsafe behavior in conjunction with multi-agent modeling, an evolutionary model of unsafe behavior of construction personnel based on risk coupling is developed in order to analyze the state change law of construction personnel and investigate the evolution path of their unsafe behavior.
4. Considering the coupling effect of influencing factors, the evolution of unsafe construction worker behavior when different control methods are chosen is simulated by modifying the control parameters. On the basis of an analysis of the simulation results of the behavioral state of construction personnel, it can be concluded that: Of all the control methods, the two-factor control method that simultaneously strengthens the management of equipment and construction personnel is the most effective in controlling the evolution of unsafe behavior among construction personnel, and control of multiple factors is superior to control of a single factor. This suggests that when controlling the evolution of unsafe behavior of construction personnel under the influence of multiple factors, it is preferable to control the coupling of multiple factors rather than a single factor, and that it is preferable to control the two-factor coupling of equipment management and construction personnel rather than all factors simultaneously. This conclusion can reduce the unnecessary consumption of human and material resources in the process of construction risk management and improve the efficacy of construction risk management in petroleum engineering construction enterprises.

The study of the coupling analysis of the factors influencing the unsafe behavior of petroleum engineering construction personnel and the evolution of their unsafe behavior control can fully investigate how to control the unsafe behaviors of construction personnel and reduce the occurrence of construction accidents by constructing a comprehensive system of safety interventions, which has theoretical significance for the risk control of petroleum engineering construction. Simultaneously, by analyzing the evolution model of unsafe behavior among construction personnel, elucidating the key factors influencing the efficacy of controlling their unsafe behavior can effectively inform the implementation of extant management protocols. This facilitates the formulation of targeted interventions for unsafe behavior among construction personnel, thereby guiding safety management practices. This approach addresses the intricacies of construction safety control processes and effectively prevents major safety incidents in petroleum engineering construction. Consequently, it holds practical significance in steering safety construction within the petroleum engineering industry. When implementing construction risk management, construction enterprises should take into account the coupling effect of risk factors, the evolution of unsafe behaviors, and the control effect of different influencing factors to select a more scientific and reasonable construction risk management method, so as to obtain better control effects and further reduce the incidence of construction accidents, which is conducive to the sustainable development of petroleum engineering construction.

### Future perspective

The utilization of a multi-agent model in this study for modeling the actual construction process introduces certain limitations, primarily stemming from model assumptions and rational simplifications of real-world scenarios. Such simplifications may create disparities between the model and actual situations, thereby compromising the accuracy of the model and the reliability of research conclusions.

In the future, research endeavors will delve deeper into the influencing factors of construction safety, encompassing external environmental factors, organizational culture, among others. Furthermore, there will be a concerted effort to explore the intricate interrelationships among different influencing factors, enhance the quality and quantity of data, and consequently improve the accuracy and predictive capabilities of the model. This will enhance the applicability of the study, facilitating the application of research findings to practical construction management. By surmounting these limitations and broadening the scope of research, the advancement of petroleum engineering construction safety studies and practices will be propelled forward, contributing to the reduction of construction accidents and the enhancement of construction safety plans.
